# Review of the Novel Echinocandin Antifungal Rezafungin: Animal Studies and Clinical Data

**DOI:** 10.3390/jof6040192

**Published:** 2020-09-28

**Authors:** Yanan Zhao, David S. Perlin

**Affiliations:** 1 Center for Discovery and Innovation, Hackensack Meridian Health, Nutley, NJ 07110, USA; david.perlin@hmh-cdi.org; 2 Department of Medical Sciences, Hackensack Meridian School of Medicine, Nutley, NJ 07110, USA

**Keywords:** rezafungin, invasive fungal infections, echinocandin, *Candida*, *Aspergillus*, *Pneumocystis*, pharmacokinetics/pharmacodynamics, efficacy, clinical trial

## Abstract

Rezafungin is a novel echinocandin drug being developed as a first-line option for treatment and prevention of invasive fungal infections. As a result of a structural modification in its parent molecule anidulafungin, rezafungin has acquired unique chemical stability conferring prolonged pharmacokinetics, as well as an administration advantage in the clinical setting compared to other drugs in the same class. Rezafungin displays potent in vitro activity against a wide spectrum of fungal pathogens, which is reflected in robust in vivo efficacy and/or pharmacodynamic studies using various animal models as well as in promising clinical trials data. This review describes in vivo characterization of rezafungin using animal models, current status of clinical development and key findings from these studies.

## 1. Introduction

Echinocandin class antifungals disrupt fungal cells via inhibition of the biosynthesis of β-1,3-D-glucan, an essential component of the fungal cell wall [1]. Given the widely reported in vitro and in vivo fungicidal efficacy against most *Candida* spp., as well as the clinical efficacy observed from the clinical settings, echinocandins are currently the recommended first-line treatment against candidemia and other invasive candidiasis [2,3]. Echinocandins are also potent against *Aspergillus* spp. Yet, since they are fungistatic instead of fungicidal, they are not used as primary treatment for invasive aspergillosis but rather as secondary or salvage therapy and sometimes in combination with antifungals from other drug classes [4,5].

**Figure 1:**
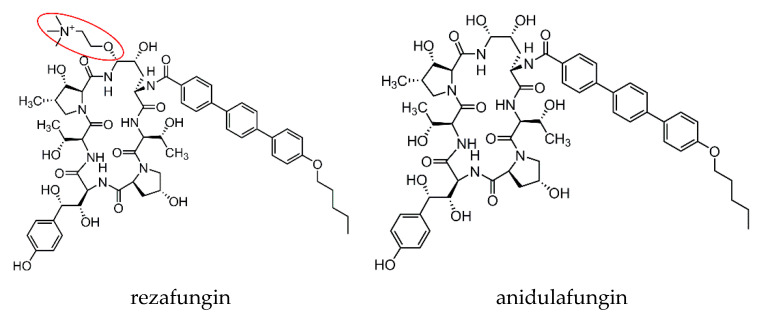
Chemical structure of rezafungin and anidulafungin. The choline amine ether at the C5 ornithine position of rezafungin is circled in red.

Rezafungin (formerly CD101) is a novel echinocandin drug currently in Phase 3 development. Its chemical structure is slightly modified from anidulafungin (Figure 1), in which the hemiaminal region at the C5 ornithine position is replaced with a choline aminal ether [6]. This slight structural modification has conferred to rezafungin an exceptional stability and enhanced solubility, which account for its largely prolonged pharmacokinetic (PK) property compared to other drugs in this class [7,8,9]. From a clinical practice standpoint, a direct benefit of the long-acting feature of rezafungin is less frequent dosing (e.g., once weekly) when compared to the daily dosing requirement for other echinocandin drugs. This dosing strategy has the potential to increase patient compliance, especially for those who require longer course of therapy after hospital discharge. The prolonged PK property also has value for prophylaxis, which has been largely dominated by triazole antifungals. This review will discuss the in vivo efficacy and pharmacokinetics and pharmacodynamics (PK/PD) of rezafungin in animal models, and clinical evidence acquired from the completed clinical trials thus far.

## 2. Preclinical Pharmacokinetics

The PK properties of rezafungin have been evaluated by several groups using a variety of animal models. In an immunocompetent mouse model of disseminated candidiasis due to *Candida albicans*, PK following single doses of intraperitoneally (IP) administered rezafungin was found to be linear over the dosing range from 10 to 60 mg/kg [10]. Drug exposure measured by plasma peak concentration (C_max_) and area under the concentration-time curve (AUC) increased dose proportionally from 23.1, 43.3, 82.3 to 95.8 μg/mL, and 736, 1250, 2380 and 3300 μg*h/mL over the course of 48 h for the doses of 10, 20, 40 and 60 mg/kg respectively. The elimination half-life was long for each dose, ranging from 29.8 to 52.0 h. Single-dose PK of rezafungin was also investigated in healthy animals [7,9]. James and colleagues performed PK analysis following a single dose of rezafungin via intravenous (IV) administration (10-min slow bolus) in healthy beagle dogs, and compared with that of anidulafungin [9]. The study found that rezafungin displayed a half-life of 53.1 h, nearly 5-fold longer than the 11.6 h measured for anidulafungin. The volume of distribution was also larger for rezafungin (1360 mL/kg) compared to 779 mg/kg for anidulafungin. Correspondingly, clearance for rezafungin was 19 mL/h/kg, lower than that for anidulafungin (47 mL/h/kg). Another study published shortly after conducted a more comprehensive single-dose PK evaluation by expanding the analysis to multiple animal species, including mice, rats, dogs and nonhuman primates (cynomolgus monkeys and chimpanzees) [7]. Authors also included both male and female animals, and different rezafungin dosing levels in their study using mice and rats. As a result, rezafungin demonstrated very consistent PK profiles, specifically, very low clearance, modest volume of distribution, and a long half-life (t_1/2_), across all species tested. PK differences between rezafungin (longer t_1/2_ and larger AUC) and anidulafungin were more pronounced in higher species (nonhuman primates).

The prolonged PK property not only sets rezafungin apart from other echinocandins in the traditional PK analysis but results in increased drug penetration at the infected tissue site. One study employed matrix-assisted laser desorption/ionization mass spectrometry imaging to investigate the spatial and quantitative distribution of rezafungin and micafungin in tissue lesions in an intra-abdominal candidiasis mouse model [11]. Drug accumulation within lesions was observed with both drugs at their humanized therapeutic doses. However, rezafungin accumulated much faster and retained remarkably longer in lesions at significantly higher levels compared to micafungin, which was associated with greater levels of tissue burden reduction and sterilization. The study also noted that rezafungin but not micafungin penetrated lesions at levels above the mutant prevention concentration of the infecting strain, suggesting the potential of rezafungin to suppress resistance development in *Candida* cells with proper dosing.

## 3. In Vivo Effectiveness

### 3.1. Animal Models

Ever since the early stage of the in vitro characterization of rezafungin, an increasing body of evidence has established that rezafungin is effective in treating invasive infections due to multiple fungal species, including *Candida* spp., *Aspergillus* spp. and *Pneumocystis* spp., in animal models [9,10,11,12,13,14,15,16,17,18,19,20,21]. Except for one study using a rabbit model to assess treatment efficacy for *Candida* endophthalmitis [21] and another one examining burden reduction in an intra-abdominal candidiasis mouse model [11], all other studies used immunocompromised mice to establish invasive infections due to different pathogens. In most cases, mice were treated with two doses of cyclophosphamide at day -4 and -1 prior to infection to deplete neutrophils, and additional doses of cyclophosphamide were given as needed to sustain persistent neutropenia for studies with longer durations such as those using survival as the endpoint. On day zero, neutropenic mice were infected with fungal strains via IV injection to establish systemic infection. Rezafungin and comparator antifungals specified in each study, as well as the vehicle control were administered at two hours post-infection, and kidney burden quantification was undertaken at a single or multiple time points at least 24 h after infection.

### 3.2. Efficacy/Pharmacodynamic Characterization in Invasive Candidiasis Model

In invasive candidiasis models, dose-dependent antifungal activity was repeatedly observed in studies that tested more than two dosing levels of rezafungin [10,13,14,17,18]. In most studies, marked in vivo fungicidal potency (≥99% killing or ≥3 log_10_ burden reduction relative to vehicle control) of rezafungin was observed with a single dose administration at dosing levels as low as 0.6 mg/kg. Good spectrum coverage of *Candida* species was obtained in these studies, and susceptibility profiles were also considered and tested as a factor of in vivo response to rezafungin. In one study [10], mice infected with a heterozygous *fks* mutant (S645P/S) *C. albicans* strain with reduced susceptibility to echinocandins responded to single doses of rezafungin across all tested dosages with almost 4 log_10_ burden reduction in kidneys compared with vehicle control at 48 h post-infection, whilst mice treated with humanized therapeutic dose of micafungin had kidney burdens 1.3~1.5 log_10_ greater than those treated with rezafungin. Similarly, a study involving an azole-resistant *C. albicans* strain induced infection showed that an over 5 log_10_ burden reduction was achieved at 48 h post-infection with a single dose treatment of rezafungin at 10 mg/kg or higher dose [14]. In a most recent study using a rabbit candidiasis model, rezafungin was found to be more effective to treat endophthalmitis caused by wild-type *C. albicans* than micafungin and voriconazole [21].

Rezafungin has also been documented by two recent studies to be effective in vivo against *C. auris* [16,19], the emerging multidrug-resistant and difficult-to-treat fungal pathogen [22,23,24]. Taking the 30~40 h half-life of rezafungin in mice into consideration, both studies employed a once every three days dosing strategy for rezafungin to mimic once-weekly dosing regimens in humans. The first study found that mice received rezafungin displayed gradual decrease in kidney fungal burdens over the treatment course [19]. The first dose of rezafungin given on day zero brought the kidney burden down by ~1 log_10_ in 24 h compared to the control, and additional doses further enlarged the burden differences between treated and untreated mice. By day seven, average burden in mice treated with rezafungin was 3.85 log_10_ lower than untreated ones. In comparison, daily amphotericin B treatment was not effective at all. Kidney burden reduction was also achieved with daily micafungin administration, however, mice in this treatment arm still had significantly higher burden (1.34 log_10_) at the study endpoint day 10 post-infection, compared to those treated with rezafungin. These efficacy results were echoed by the second study, in which four *C. auris* strains with rezafungin MIC ranging from 0.06 to 2 µg/mL were included in the evaluation [16].

The pharmacodynamic driver of rezafungin to treat invasive candidiasis has been elucidated through a series of systematic evaluation [16,17,18,25]. The first study examined the pharmacodynamic activity of rezafungin against select *C. albicans*, *C. glabrata*, and *C. parapsilosis* strains with different in vitro susceptibility profiles to echinocandins [17]. Over a 4-fold dose range from 0.25 to 64 mg/kg, rezafungin demonstrated a dose-dependent kidney burden reduction activity. The best PK/PD index associated with in vivo efficacy is the ratio of AUC over 24 h in the steady state to the minimum inhibitory concentration (MIC) (AUC/MIC). The median stasis 24-h free-drug AUC/MIC targets were 2.92 for *C. albicans*, 0.07 for *C. glabrata*, and 2.61 for *C. parapsilosis*; and the targets for 1-log_10_ kill endpoint were two- to four-fold higher. It was noted that these PK/PD targets were numerically lower for all three species than those of other echinocandins. Based on the dosage determined by Phase 1 clinical studies [8], the stasis target would be expected to be achieved against both *C. albicans* and *C. parapsilosis* isolates with MICs of ≤1 µg/mL and against all *C. glabrata* isolates with MICs of ≤16 µg/mL. The same group assessed the pharmacodynamic activity of rezafungin against *C. auris*, *C. tropicalis* and *C. dubliniensis* in two subsequent studies [16,18]. AUC/MIC was confirmed to be the best PD predictor in both studies. The stasis free-drug 24-h AUC/MIC target was 1.88 for *C. auris*, 11.65 for *C. tropicalis*, and 9.53 for *C. dubliniensis*, and the 1-log_10_ kill target was 5.77, 20.92, and 32.15 for these three species, respectively. Based on contemporary in vitro surveillance susceptibility data [26,27,28], these PK/PD targets are likely to be exceeded for >90% of *C. auris* isolates, and >99% of isolates of *C. tropicalis* and *C. dubliniensis*, with the previously studied human dose of 400 mg administered IV once weekly. Further, data from dose-fractionation studies suggested that the in vivo anti-*Candida* efficacy of rezafungin is not only driven by AUC/MIC, but influenced by the shape of the exposure curve [25]. A single dose administration of rezafungin was associated with a greater degree of fungal killing than the same dose divided into twice weekly or daily regimens over seven days.

### 3.3. In Vivo Efficacy against Other Fungal Pathogens

Given the highly favorable PK property and potent in vitro activities of rezafungin, a few studies have been undertaken to evaluate the potential of rezafungin as alternative therapeutic option or prophylaxis for invasive infections caused by *Aspergillus fumigatus* and *Pneumocystis murina* [12,13,14,15,20]. Using an immunocompromised mouse model of disseminated invasive aspergillosis, Ong and colleagues showed for the first time that consecutive 5-day rezafungin treatment initiated 24 h post-inoculation significantly increased 10-day survival of infected mice from 20% with vehicle control to 80%, 90%, and 100% with rezafungin at 0.2, 1, and 5 mg/kg, respectively [13]. Later, the authors expanded their evaluation by introducing dose-fractionation in rezafungin regimens, wherein rezafungin was administered as a single dose at 2 mg/kg or fractionated at 0.2 mg/kg twice daily for 5 days. As a result, both single dose and twice-daily fractionated rezafungin regimens successfully protected mice from death over the 10-day study course [14]. Another recent study employed extended-interval dosing of rezafungin (1, 4, and 16 mg/kg on days 1, 4, and 7 post-inoculation) to treat disseminated invasive aspergillosis caused by azole-resistant *A. fumigatus* (TR34/L98H mutant) [15]. Significantly improved survival was achieved with all rezafungin arms, as with supra-therapeutic posaconazole (20 mg/kg twice daily). Kidney fungal burden, as measured by quantitative real-time PCR, was also significantly reduced in mice treated with rezafungin although variability was observed.

It has been reported that currently approved echinocandins are not suitable for monotherapy to treat *Pneumocystis* pneumonia (PCP) [29]. However, the remarkably prolonged PK of rezafungin makes it a highly favorable option for prophylaxis. Using an immunocompromised mouse model of PCP, a 6-week study found that 3-week prophylactic rezafungin had comparable efficacy to the PCP standard of care trimethoprim/sulfamethoxazole (TMP/SMX) in preventing the development of PCP by blocking the formation of reproductive forms (trophic and cyst/asci) of *P. murina* [12]. Most recently, the study group further extended the evaluation time window to a maximum of 14 weeks, to address whether *Pneumocystis* infection in a similar immunosuppressed mouse model could re-activate in six weeks after two to eight weeks of prophylactic therapy using different dosing regimens of rezafungin [20]. The study found that as short as four weeks rezafungin prophylaxis was effective to prevent *P. murina* organisms from activating infection after cessation of therapy. Significant survival benefit was also observed with rezafungin compared to caspofungin, when both drugs were administered at their own humanized therapeutic dose with the same dosing frequency.

A summary of in vivo characterization of rezafungin is provided in Table 1.

## 4. Clinical Development

Thus far, rezafungin has progressed to Phase 3 studies. The most updated information of the clinical trials is summarized in Table 2.

Phase 1 evaluation of rezafungin consisted of two randomized, double-blind, placebo-controlled, dose-escalation studies, the single-ascending-dose study (ClinicalTrials.gov identifier: NCT02516904) and the multiple-ascending-dose study (NCT02551549), completed in October 2015 and January 2016, respectively. The primary objective of these studies was to determine the safety and pharmacokinetics of IV (infusion over 1 h) administered rezafungin in healthy adults [8]. A total of 32 subjects in the single-ascending-dose study were randomized to four dose cohorts (50, 100, 200, 400 mg) of eight subjects each (six active and two placebo) and received one-time drug treatment. In the multiple-ascending-dose study, 24 participants were randomized to three dose cohorts (100 mg × 2 doses, 200 mg × 2 doses, and 400 mg × 3 doses) of eight subjects each (six active and two placebo) to receive once weekly treatment until completion of the regimen determined by the dose cohort. No safety issues were noted concerning abnormal blood laboratory results, electrocardiograms, vital signs, or physical exams. There were no serious or severe adverse events, or withdrawals from the study due to an adverse event. The majority of the adverse events were mild, and all completely resolved prior to the end of the study. Mild transient infusion reactions such as flushing, nausea and chest tightness were seen with the third 400 mg dose in the multiple-dose study. Upon PK analyses, rezafungin showed dose-proportional plasma exposures (both AUC and C_max_) and low apparent clearance (<0.28 L/h) in both studies. A substantially longer half-life than any currently available echinocandins was noted in both studies, with ~80 h following the first dose and ~150 h following the second or third dose. Altogether, the Phase 1 clinical data demonstrated good safety and favorable PK properties of rezafungin and established appropriate dosing regimens for the Phase 2 studies.

Following Phase 1 studies, two Phase 2 clinical trials (STRIVE and RADIANT) involving different formulations (IV and topical) of rezafungin with different indication purposes were launched almost simultaneously in 2016. STRIVE (NCT02734862) was a global, Phase 2, randomized and double-blind clinical trial, aimed to systematically evaluate the safety, tolerability, and efficacy of IV infused rezafungin compared to caspofungin in the treatment of candidemia and/or invasive candidiasis. STRIVE was conducted in two consecutive parts, Part A and Part B, and a total of 207 patients were enrolled in both Europe and North America. Adults (≥18 years) with mycologically confirmed candidemia and/or invasive candidiasis were randomized (1:1:1) to receive rezafungin IV for up to four weeks dosed at either 400 mg weekly (Group 1) or 400 mg on week one and 200 mg weekly thereafter (Group 2), or standard of care (SOC; daily caspofungin with optional criteria-defined oral stepdown fluconazole after ≥ three days of IV therapy; Group 3). The primary safety and tolerability endpoints were treatment-emergent adverse events (TEAEs) through days 45–59; and the primary efficacy endpoint was overall success at day 14 demonstrated by mycological eradication and clinical cure. According to the early analysis of Part A results and the latest wrap-up report for both Part A and B [30,31], rezafungin displayed good safety, with TEAE incidence comparable to that in the SOC group. Rezafungin 400 mg/200 mg once weekly demonstrated greater efficacy than SOC, with highest rates of overall success on day 14 (60.5% for RZF 400 mg, 76.1% for RZF 400 mg/200 mg, and 67.2% for SOC) and the lowest rate of 30-day all-cause mortality (15.8% for RZF 400 mg, 4.4% for RZF 400 mg/200 mg, and 13.1% for SOC) across all treatment arms. These results supported further Phase 3 evaluation of rezafungin 400 mg/200 mg regimen to treat candidemia and invasive candidiasis.

The second Phase 2 study, RADIANT (NCT02733432), was designed to evaluate the safety, tolerability, and potential efficacy of topical formulations of rezafungin for the treatment of moderate to severe episodes of acute vulvovaginal candidiasis [32]. A total of 126 patients were enrolled in this study and randomized to three treatment cohorts: rezafungin 3% gel applied intravaginally on days one and two (cohort 1), rezafungin 6% ointment applied intravaginally on day one (cohort 2), and oral fluconazole 150 mg on day one (cohort 3). Primary outcomes of clinical and mycological cure, as demonstrated by changes in vaginal scores and mycological cultures, were assessed on day 7 (±2 days), day 14 (±2 days), and day 28 (±7 days). While both topical formulations of rezafungin were safe and well tolerated, the study found that rates of clinical and mycological cure produced by topical rezafungin regimens were numerically although not significantly lower than that achieved with fluconazole. These results suggest that the studied formulations of rezafungin are yet to be a valid alternative to current standard of care. Future studies of topical rezafungin would need to evaluate other formulations that either increase or prolong vaginal exposure to the active medication or possibly even higher concentrations.

Despite the topical formulation constraint, promising results observed in the STRIVE trial warranted progression to Phase 3 evaluation of IV rezafungin for candidemia and invasive candidiasis. Currently, there are two Phase 3 clinical trials ongoing. The first pivotal study ReSTORE (NCT03667690) which started patient enrollment in October 2018 is a multicenter, randomized, double-blind study examining rezafungin 400 mg/200 mg regimen for the treatment of candidemia and invasive candidiasis. The active comparator in ReSTORE is caspofungin 70 mg IV loading dose followed by 50 mg IV once daily for 14~28 days. The primary endpoint will be 30-day all-cause mortality and 14-day global cure measured by clinical, radiological, and mycological indices. Another ongoing Phase 3 trial ReSPECT (NCT04368559) is to evaluate rezafungin for the prevention of invasive fungal infections caused by *Candida*, *Aspergillus* and *Pneumocystis* in patients undergoing allogeneic blood and marrow transplantation. Over 460 patients are expected to be enrolled and randomized to one of two prophylactic arms: rezafungin 400 mg loading dose in week 1 followed by 200 mg once weekly for a total of 13 weeks (group 1), or standard azole prophylaxis (oral fluconazole or posaconazole) and anti-PCP prophylaxis (oral TMP/SMX) for 13 weeks (group 2). Both noninferior and superior 90-day fungal-free survival will be evaluated as primary outcome.

## 5. Conclusions

Rezafungin is a novel echinocandin with prolonged PK properties compared to other drugs in the same class. With robust preclinical evidence accumulated in animal models and promising results released from Phase 2 STRIVE trial, rezafungin has entered two Phase 3 clinical studies, to evaluate the efficacy of IV regimens for the treatment of candidemia and invasive candidiasis, and for the prevention of invasive fungal infections caused by *Candida* spp., *Aspergillus* spp., and *Pneumocystis* spp. Rezafungin, with its potent antifungal activities and unique pharmacological properties, holds the potential as a next-generation first-line antifungal treatment and prophylaxis option over current standards of care to address invasive fungal infections.

## Figures and Tables

**Table 1:** In vivo characterization of rezafungin.

Aspect	Animal Model	Feature
PK/PD	Healthy mouse, rat, dog, cynomolgus monkey, chimpanzee; Immunocompetent mouse model of invasive candidiasis; Immunocompetent mouse model of intra-abdominal candidiasis; Neutropenic mouse model of invasive candidiasis	Dose proportional drug exposure (C_max_ and AUC); Very long half-life (longer than any of currently approved echinocandin drug); Low clearance and wide tissue distribution; Quick and sustained penetration at infected tissue sites; AUC/MIC is the best index associated with efficacy; The shape of exposure curve also influences efficacy
Efficacy	Neutropenic mouse model of invasive candidiasis; Immunocompetent mouse model of intra-abdominal candidiasis; Immunocompetent rabbit model of invasive candidiasis; Neutropenic mouse model of disseminated invasive aspergillosis; Immunosuppressed mouse model of *Pneumocystis* pneumonia	Comparable or better efficacy than comparator drug (anidulafungin or micafungin) in *Candida* infection models, including those caused by echinocandin- and azole-resistant *Candida* strains; Effective in improving survival and reducing kidney burdens in both azole-sensitive and -resistant *Aspergillus* infections; Comparable efficacy to the standard of care (TMP/SMX) in prevention of *Pneumocystis* pneumonia

**Table 2:** Summary clinical evaluation of rezafungin.

Clinical Status	Trial (ClinicalTrials.gov Identifier)	Objective	Key Finding
Phase 1 (completed)	Single-ascending-dose study (NCT02516904)	Safety, tolerability, and PK	No safety issues were noted; Dose-proportional plasma exposures (AUC and C_max_) and low clearance; Long half-life (~80 h after first dose and ~150 h following addition dose)
	Multiple-ascending-dose study (NCT02551549)		
Phase 2 (completed)	STRIVE (NCT02734862)	Efficacy to treat candidemia and invasive candidiasis	Rezafungin IV 400 mg first week followed by 200 mg once weekly regimen showed greater efficacy than caspofungin
	RADIANT (NCT02733432)	Efficacy to treat vulvovaginitis	Topical formulations of rezafungin were safe and well tolerated; Cure rates of topical rezafungin were lower than those achieved with fluconazole
Phase 3 (ongoing)	ReSTORE (NCT03667690)	Efficacy to treat candidemia and invasive candidiasis	To be determined
	ReSPECT (NCT04368559)	Efficacy to prevent invasive fungal infections due to *Candida*, *Aspergillus*, and *Pneumocystis*	To be determined
